# Associations of Platelet Count with Inflammation and Response to Anti-TNF-α Therapy in Patients with Ankylosing Spondylitis

**DOI:** 10.3389/fphar.2020.559593

**Published:** 2020-11-06

**Authors:** Hongyan Qian, Rongjuan Chen, Bin Wang, Xiaoqing Yuan, Shiju Chen, Yuan Liu, Guixiu Shi

**Affiliations:** ^1^ Department of Rheumatology and Clinical Immunology, The First Affiliated Hospital of Xiamen University, Xiamen, China; ^2^ School of Medicine, Xiamen University, Xiamen, China; ^3^ Ningbo City Medical Treatment Center Lihuili Hospital, Ningbo, China

**Keywords:** ankylosing spondylitis, platelet count, anti-TNF-α therapy, treatment outcomes, biomarkers

## Abstract

**Background: **Increased platelet count has been reported in ankylosing spondylitis (AS) patients, but its clinical significance is still largely elusive. The objective of this study was to evaluate the clinical role of platelet count in AS patients, especially its impact on treatment outcomes.

**Methods:** A case-control study containing 35 AS patients receiving anti-tumor necrosis factor-α (anti-TNF-α) therapy and 45 healthy controls was performed, and AS patients were followed at least 6 months after anti-TNF-α therapy. A systematic review and meta-analysis of studies containing relevant data on outcomes of interest was also performed.

**Results: **AS patients had significantly higher platelet count than controls (*p* = 0.0001), and the significantly increased platelet count in AS patients was confirmed in a meta-analysis of 14 studies involving 1,223 AS patients and 913 controls (mean difference = 39.61, 95% CI 27.89–51.34, *p* < 0.001). Besides, platelet count was significantly correlated with ESR (*p* < 0.001) and was moderately correlated with ASDAS-CRP score (*p* = 0.002). Moreover, anti-TNF-α therapy could reduce platelet count in AS patients at the first month and the effect was maintained through the treatment duration. In the prospective follow-up study of those 35 AS patients, those responders to anti-TNF-α therapy had significantly lower platelet count than nonresponders (*p* = 0.015). Logistic regression analysis suggested that lower platelet count was associated with higher possibility of achieving good response to anti-TNF-α therapy in AS patients (odds ratio = 2.26; 95% CI = 1.06–4.82; *p* = 0.035).

**Conclusion:** This study suggested that platelet count was associated with inflammation severity and treatment outcomes in AS patients, and elevated platelet count was a promising biomarker of poorer response to anti-TNF-α therapy. The findings above need to be validated in more future studies.

## Introduction

Ankylosing spondylitis (AS) is a common rheumatic disease characterized by chronic low back pain and spinal ankylosing (Sieper and Poddubnyy, 2017). AS can impair spinal function and reduce life quality, which has caused serious harm to public health (Macfarlane et al., 2020). Current treatment for AS patients mainly includes nonsteroidal anti-inflammatory drugs (NSAIDs) and biological disease-modifying antirheumatic drugs (DMARDs), and anti-tumor necrosis factor-α (anti-TNF-α) agents are the most commonly used biological DMARDs (Sieper and Poddubnyy, 2016). Although biological DMARDs have largely improved the treatment outcomes of AS patients, nearly half of the patients fail to achieve a good treatment response and are at high risk of clinical progression (Ornbjerg et al., 2019). Biological DMARDs such as anti-TNF-α therapy can result in increasing risk of infections including tuberculosis (Atzeni et al., 2019). To reduce infections risk and promote treatment outcomes, personalized treatment strategy based on the clinical characteristics of individual AS patient is necessary (Poddubnyy and Sieper, 2019). Current knowledge regarding the predictors of treatment outcomes in AS patients is still limited, and more studies on the promising biomarkers of predicting treatment response to biological DMARDs in AS patients are needed.

Despite the roles of platelets in hemostasis and thrombosis, their roles in regulating inflammation and immunity have gained increasing attentions in recent years (Maurer et al., 2020; Rayes et al., 2020; van der Poll and Parker, 2020). Platelets have been recognized as crucial regulators of inflammatory processes under various pathophysiological conditions (Karhausen et al., 2020). The inflammation caused by overactivated platelets can further promote the development of thrombosis, atherosclerosis, and cardiovascular diseases (Barrett et al., 2019; Garshick et al., 2020; Schnorbus et al., 2020). Platelets exert critical roles in the development of inflammatory diseases such as inflammatory bowel disease (IBD) (Chimen et al., 2019; Petrey et al., 2019; Nicolai and Massberg, 2020). Some studies have uncovered that platelets have important roles in the development of rheumatic diseases such as rheumatoid arthritis (RA) and systemic lupus erythematosus (SLE) (Andrianova et al., 2019; Olumuyiwa-Akeredolu et al., 2019; Tessandier et al., 2020). Although there are some published studies that have assessed the clinical significance of platelets in AS patients, no definite conclusion is available up to now. In light of these undefined questions, the objective of this study was to evaluate the clinical role of platelet count in AS patients, especially its impact on treatment outcomes.

## Materials and Methods

### Patients and Treatment

Thirty-five patients with active AS and treated with anti-TNF-α agents were consecutively enrolled into the study. All AS patients were diagnosed by modified New York classification criteria (van der Linden et al., 1984; Raychaudhuri and Deodhar, 2014). To be enrolled in this study, AS patients must have a Bath Ankylosing Spondylitis Disease Activity Index (BASDAI) score of more than 1 and no history of biological DMARDs such as anti-TNF-α agents and anti-IL-6 agents. This study was performed between September 2018 and June 2019, and 35 AS patients were finally enrolled. The mean age was 33.1 ± 8.8 years, and the mean disease duration was 8.7 ± 5.1 years. AS patients were followed at least 6 months after the initial treatment of anti-TNF-α agents, and clinical and laboratory assessments were performed at baseline and at each follow-up. Forty-five healthy controls were recruited from the medical examination center in our hospital. The study protocol was approved by the Ethics Committee of our hospital, and written informed consent was obtained from all participants.

### Clinical Assessment and Laboratory Testing

Laboratory parameters such as C-reactive protein (CRP), erythrocyte sedimentation rate (ESR), and platelet count were tested in the Laboratory Department of our hospital. All participants underwent detailed physical examination, and clinical data such as demographic data, Ankylosing Spondylitis Disease Activity Score (ASDAS)-CRP, and BASDAI score were obtained by interview and medical records. The treatment response was evaluated by BASDAI, and patients with an improvement of no less than 50% in BASDAI score at 6 months were defined as responders.

### Outcomes of Interest

The primary outcome of interest was the influence of platelet count on treatment response of anti-TNF-α therapy in AS patients. The secondary outcomes of interest were the difference in platelet count between AS patients and controls, the correlations of platelet count with disease activity, and the impact of anti-TNF-α therapy on the platelet count in AS patients.

### Statistical Analysis

In the sample size calculation of the analyses regarding the difference or changes in platelet count, to detect a difference with a power of 80% at 95% confidence level (95% CI), the sample size required will be 30 in each group if a desired mean difference is 40 and the standard deviation (SD) is 55. In the analysis of correlations of platelet count with disease activity markers of AS patients, to detect a difference with a power of 80% at 95% CI, the sample size required will be 36 if a desired correlation coefficient was 0.40. In this study, the number of AS patients was 35 and the number of controls was 45, which led to a power of 89.7% in the analyses regarding the difference or changes in platelet count, and a power of 78.4% in the analysis of correlations of platelet count with disease activity markers. The power for the analysis of the relationship between platelet count and treatment response was likely to be low because the number of AS patients was only 35.

Data were shown as either mean ± SD or median with quartiles, and differences were assessed by Student *t*-test or Mann–Whitney *U* test. Correlation was determined by Pearson correlation test. Receiver operating characteristic (ROC) analysis and logistic regression analysis were performed to evaluate the impact of platelet count on response to anti-TNF-α treatment. Only those noncollinear variables that were correlated with platelet count were adjusted by multivariate logistic regression analysis. STATA (Version 12.0, StataCorps, TX, USA) was used, and two-sided *p* values less than 0.05 were considered statistically significant.

### Systematic Review and Meta-Analysis

To validate the findings in our study, a systematic review and meta-analysis was performed according to Preferred Reporting Items for Systematic reviews and Meta-Analyses (PRISMA) (Liberati et al., 2009). A literature search in PubMed was performed in March 2020 to find studies on the roles of platelet count in AS patients. An updated literature search was performed on September 2, 2020, to identify additional studies. The following search strategy was used: (ankylosing spondylitis or axial spondyloarthritis or spondyloarthritis) and (platelet or platelets). To be included into the meta-analysis, studies must meet the following eligible criteria: 1) clinical observational studies using cohort, cross-sectional, or case-control design; 2) patients were AS patients, and controls were those without AS; 3) assessing the difference in platelet count between AS patients and controls, or the impact of anti-TNF-α therapy on platelet count in AS patients, or the correlations of platelet count with disease activity, or the influence of platelet count on treatment response toward anti-TNF-α therapy in AS patients; and 4) reporting data that could be integrated by meta-analysis. Studies containing duplicated data or without usable data on outcomes of interest were excluded. Two authors decided on the inclusion or exclusion of studies, and discrepancy was settled by discussion among all authors.

Outcomes of interest were the influence of platelet count on treatment response to anti-TNF-α therapy, the difference in platelet count between AS patients and controls, and the correlations of platelet count with disease activity. Information on key characteristics of included studies such as the first author, publication year, types of treatment, and outcomes were extracted from each study. The quality of those included studies was reviewed through three items including the selection of AS patients and controls, the comparability between exposed individuals and nonexposed controls, and the assessment of outcomes, which mainly followed Newcastle–Ottawa Scale (NOS) (Stang, 2010). Studies with NOS score of 6 or more were deemed to have good quality, whereas those with NOS score of 5 or less were deemed to have suboptimal quality.

Risk estimates such as odds ratios (ORs) or mean differences with 95% CIs were pooled using meta-analysis. The between-study variance of included studies was evaluated using *I* ^2^ method, and *I* ^2^ more than 50% suggested the existence of high heterogeneity (Higgins et al., 2003). To reduce the possible impact of heterogeneity, data were pooled using random-effect meta-analysis (DerSimonian and Laird, 1986). Subgroup analysis by study quality was performed. To analyze the relationship between platelet count and BASDAI score, we further performed a meta-analysis of the difference in platelet count between AS patients with high disease activity (BASDAI ≥ 4) and those with low disease activity (BASDAI < 4). To determine whether BASDAI score at the time of enrollment was associated with the correlation between platelet count and BASDAI score, meta-regression was performed. STATA (Version 12.0, StataCorps, TX, USA) was used in meta-analysis. A *p* value < 0.05 was considered statistically significant.

## Results

### Significantly Increased Platelet Count in AS Patients

In our case-control study, the platelet count in AS patients before anti-TNF-α therapy was significantly higher than that in controls (300.7 × 10^9^/L vs. 251.4 × 10^9^/L; *p* = 0.0001) (Supplementary Table S1).

The process of study selection in the meta-analysis was shown in Supplementary Figure S1. Through the initial literature search, 141 articles were retrieved, 114 articles were excluded for obviously irrelevant study design or being reviews, and 27 studies were evaluated by reading full-text (Supplementary Figure S1). Fifteen studies were further excluded for the lack of usable data, and two additional studies were identified though an updated literature search on September 2, 2020 (Supplementary Figure S1). Therefore, 14 published observational studies were finally included (Kisacik et al., 2008; Wang et al., 2008; Yazici et al., 2010; Nalbant et al., 2011; Orum et al., 2012; Boyraz et al., 2014; Bozan et al., 2016; Sezgin et al., 2017; Huang et al., 2018; Tang et al., 2018; Enginar and Kacar, 2019; Al-Osami et al., 2020; Liu et al., 2020; Zhuang et al., 2020). Supplementary Table S2 showed the main characteristics of those observational studies included in the meta-analysis. Most of those studies recruited AS patients who had been diagnosed according to the modified New York criteria for AS, and the controls were healthy controls without disease history of rheumatic diseases such as AS (Supplementary Table S2). Therefore, the enrollment criteria for participants among those included studies were similar. Quality assessment suggested most studies had good quality (Supplementary Table S3).

**FIGURE 1:**
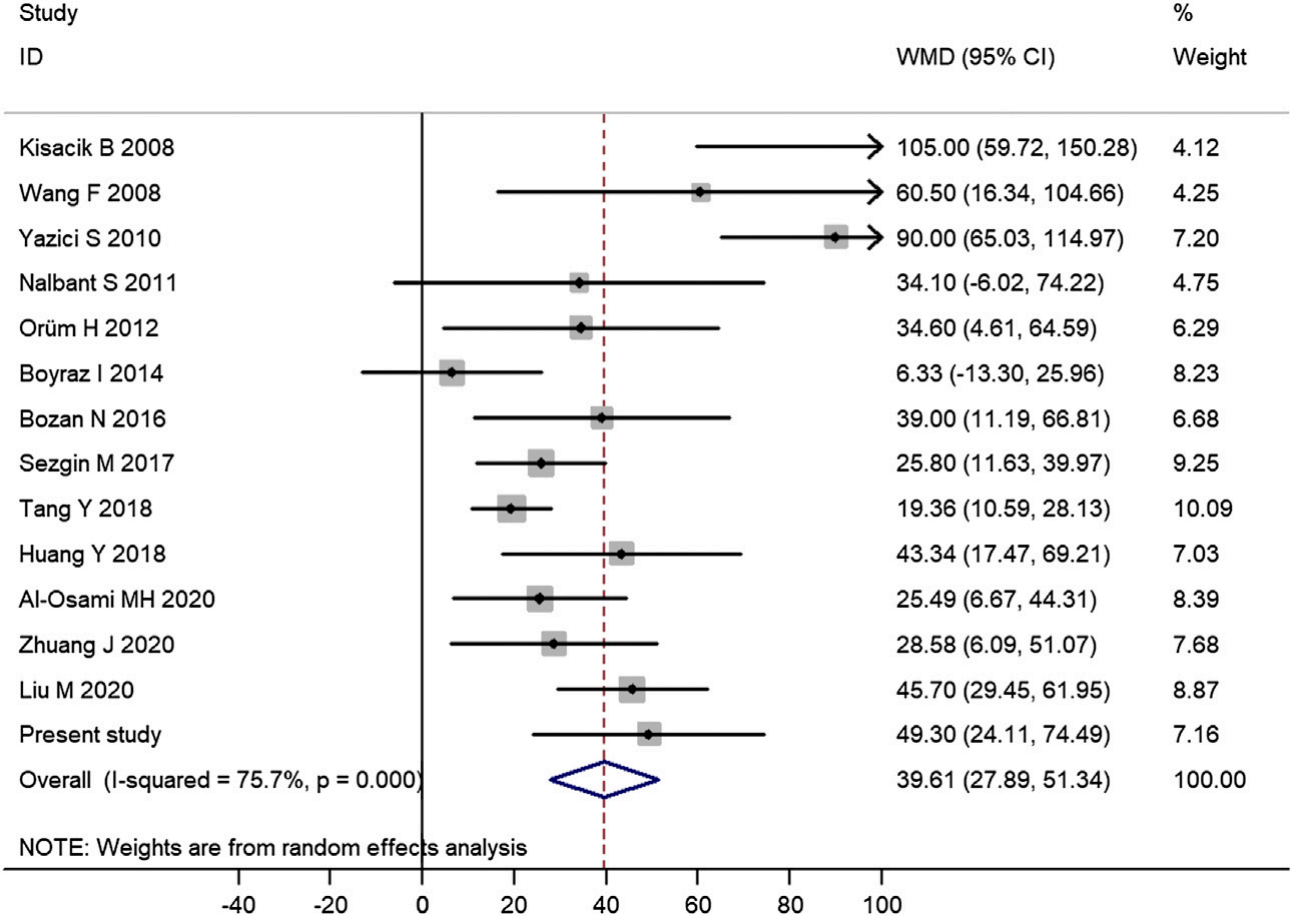
Meta-analysis of 14 studies confirmed the significantly increased platelet count in AS patients. (WMD, weighted mean difference; 95% CI, 95% confidence interval. Black circle represents the WMD for each study, and the solid horizontal line across the square represents 95% CI for each study. The size of the gray box represents the weight for each study, and it is proportional to the sample size of each study. The diamond represents the pooled WMD, and its left and right vertices represent the 95% CI.)

Among those 14 published studies, 13 studies provided data on the difference of platelet count between AS patients and controls (Supplementary Table S2). Therefore, there were a total of 14 studies including this study assessing the difference of platelet count between AS patients and controls, which contained a total of 1,223 AS patients and 913 controls. Meta-analysis of those data confirmed the significantly increased platelet count in AS patients (mean difference = 39.61, 95% CI 27.89–51.34, *p* < 0.001) (Figure 1). Subgroup analysis of 12 studies with good quality further confirmed a significantly increased platelet count in AS patients (mean difference = 35.45, 95% CI 24.26–46.64, *p* < 0.001) (Supplementary Figure S2).

### Correlations of Platelet Count with Disease Activity of AS Patients

**FIGURE 2:**
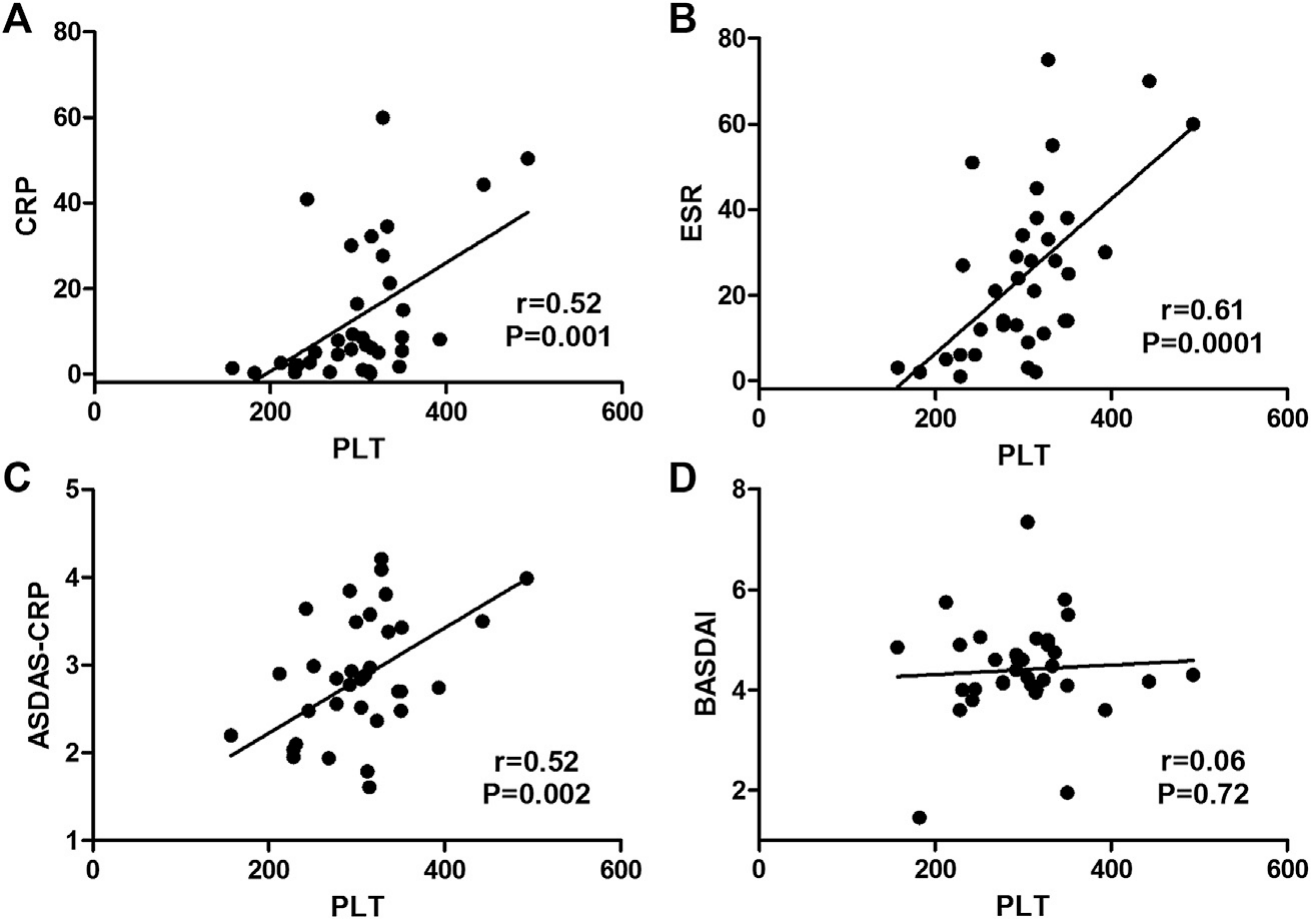
Correlations of platelet count with disease activity markers in AS patients. (Platelet count had a moderate correlation with CRP **(A)** and ASDAS-CRP score **(C)** and had a significant correlation with ESR **(B)**, but it was not correlated with BASDAI score **(D)**.)

Among those 35 AS patients, platelet count had a significant correlation with ESR (coefficient *r* = 0.61, *p* = 0.0001), and had a moderate correlation with CRP (coefficient *r* = 0.52, *p* = 0.001) and ASDAS-CRP score (coefficient *r* = 0.52, *p* = 0.002), but it was not correlated with BASDAI score (coefficient *r* = 0.06, *p* = 0.72) (Figure 2).

In the systematic review, there were only four published studies reporting outcomes on the correlations of platelet count with disease activity of AS patients (Yazici et al., 2010; Sezgin et al., 2017; Enginar and Kacar, 2019; Liu et al., 2020). The clinical characteristics of AS patients in those four published studies and this study were shown in Supplementary Table S4. AS patients from the study by Yazici et al. (2010) had higher disease activity than those from this study (Supplementary Table S4).

**FIGURE 3:**
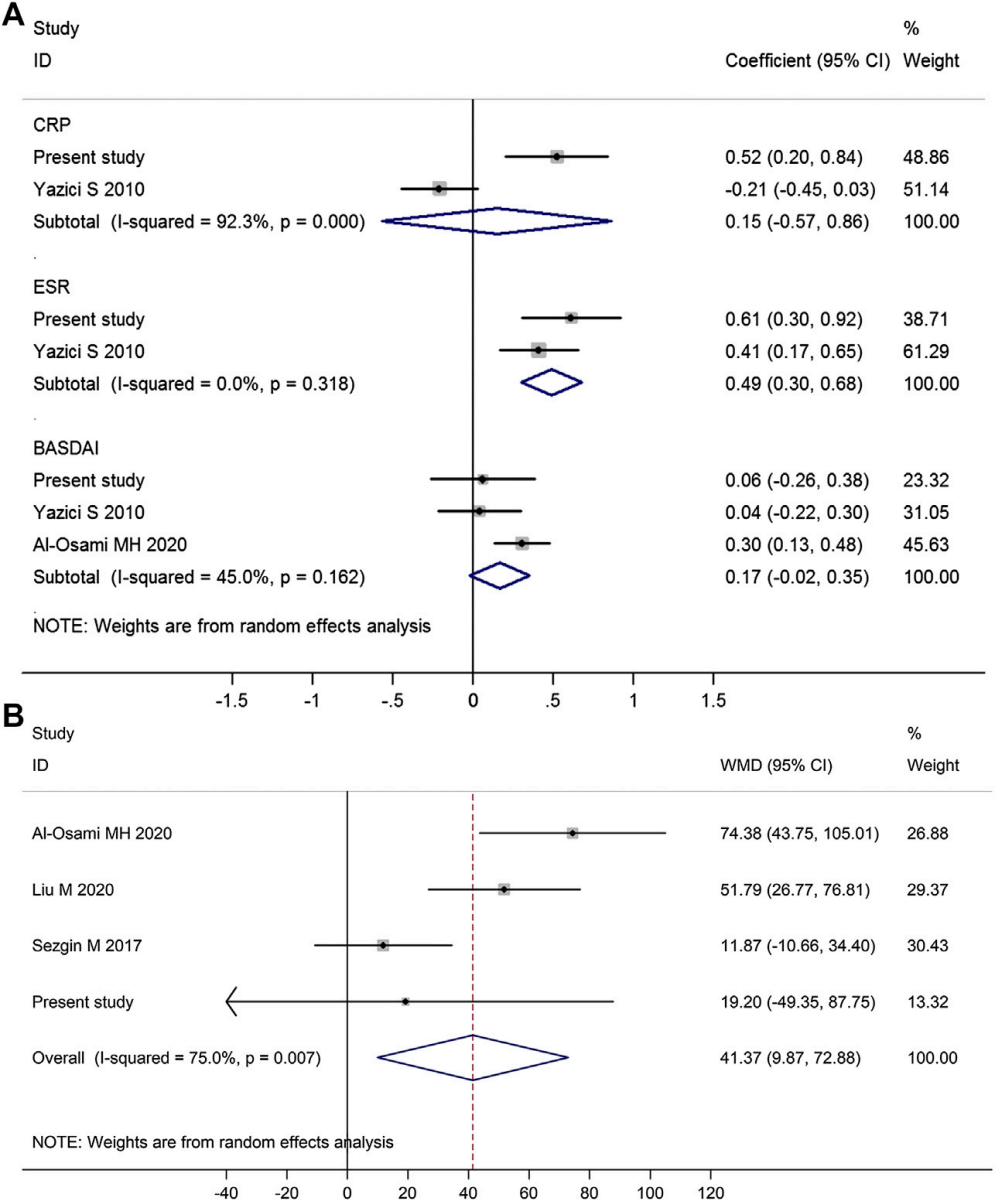
Forest plot in the meta-analysis of the correlations between platelet count and disease activity markers in AS patients. (Meta-analysis suggested that platelet count was positively associated with ESR but not BASDAI score or CRP **(A)**, and AS patients with high disease activity (BASDAI ≥ 4) had significantly higher platelet count than those with low disease activity (BASDAI < 4) **(B)**. WMD, weighted mean difference; 95% CI, 95% confidence interval. Coefficient represents the correlation coefficient between platelet count and disease activity parameters. The diamond represents the pooled correlation coefficient, and its left and right vertices represent the 95% CI.)

Meta-analysis of those studies suggested that platelet count was positively correlated with ESR (pooled correlation coefficient = 0.49, 95% CI 0.30–0.68, *p* < 0.001), but was not significantly correlated with CRP (pooled correlation coefficient = 0.15, *p* = 0.69) (Figure 3A).

There were three studies reporting data on the correlation between platelet count and BASDAI score, and meta-analysis of those three studies suggested platelet count was not significantly correlated with BASDAI score (pooled correlation coefficient = 0.17, 95% CI −0.02 to 0.35, *p* = 0.09; Figure 3A). Meta-regression analysis showed that BASDAI score at the time of enrollment was not significantly associated with the correlation between platelet count and BASDAI score (*p* = 0.382; Supplementary Figure S3). Four studies reporting data on the difference in platelet count between AS patients with high disease activity (BASDAI ≥ 4) and those with low disease activity (BASDAI < 4), and meta-analysis of those four studies suggested that AS patients with high disease activity (BASDAI ≥ 4) had significantly higher platelet count than those with low disease activity (BASDAI < 4) (mean difference = 41.37, 95% CI 9.87–72.88, *p* = 0.010) (Figure 3B).

Only this study assessed the correlation of platelet count with ASDAS-CRP of AS patients and identified a positive correlation (correlation coefficient = 0.52, *p* = 0.002) (Figure 2).

### Changes of Platelet Count After Anti-TNF-α Therapy

**FIGURE 4:**
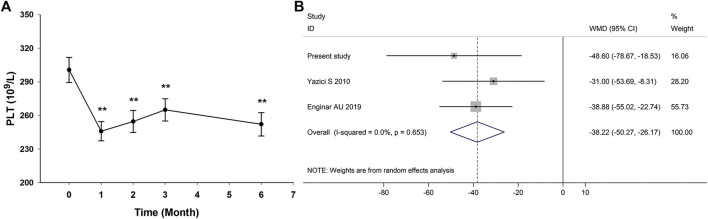
Impact of anti-TNF-α therapy on platelet count in AS patients. (Anti-TNF-α therapy could reduce platelet count in AS patients at the first month and the effect was maintained through the treatment duration **(A)**. Meta-analysis confirmed the significantly reduced platelet count in AS patients after anti-TNF-α therapy **(B)**. WMD, weighted mean difference; 95% CI, 95% confidence interval.)

In the prospective follow-up study of 35 AS patients, anti-TNF-α therapy could reduce platelet count in AS patients at the first month and the effect was maintained through the treatment duration (Figure 4A; Supplementary Table S5). There were only two published studies reporting data on the impact of anti-TNF-α therapy on platelet count in AS patients (Yazici et al., 2010; Enginar and Kacar, 2019). The study by Yazici et al. (2010) reported the change of platelet count after anti-TNF-α therapy for 3 months, and the study by Enginar and Kacar (2019) reported the change of platelet count after anti-TNF-α therapy for at least 6 months. Meta-analysis of those two studies and this study confirmed the significantly reduced platelet count in AS patients after anti-TNF-α therapy (pooled mean difference: −38.22; 95% CI −50.27 to −26.17; *p* < 0.001) (Figure 4B).

### Platelet Count and Treatment Response to Anti-TNF-α Therapy

**FIGURE 5:**
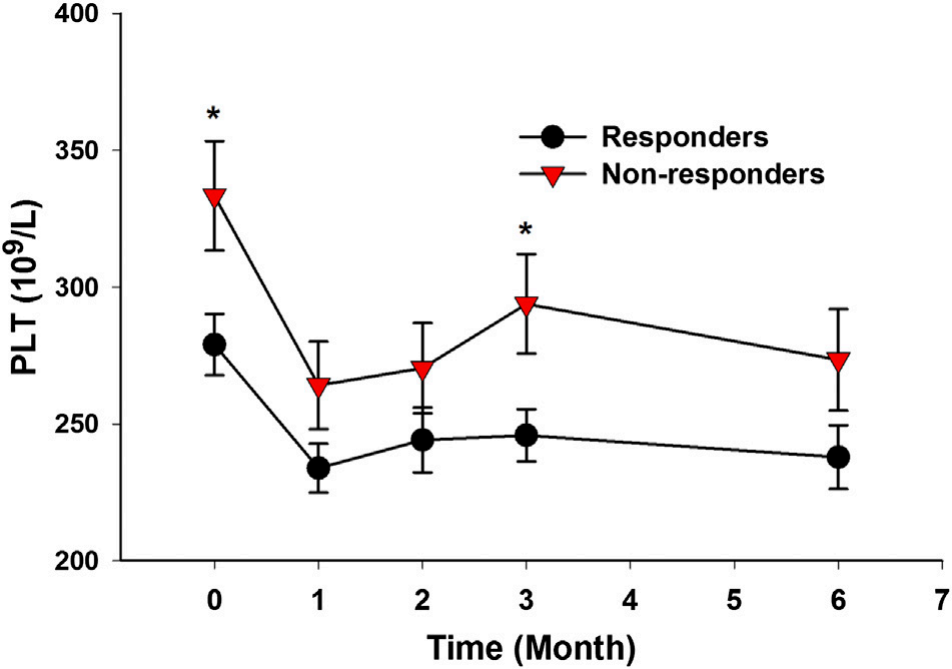
AS patients with good response to anti-TNF-α therapy had lower platelet count. (Responders to anti-TNF-α therapy had significantly lower platelet count at baseline and at 3 months after treatment (^*^ *p* < 0.05).)

In the prospective follow-up study of those 35 AS patients, 21 patients (60.0%) had an improvement of no less than 50% in BASDAI score at 6 months after anti-TNF-α therapy. The differences in baseline clinical characteristics between responders and nonresponders were shown in Table 1. Compared with nonresponders, responders had lower ESR level (*p* = 0.035), lower CRP level (*p* = 0.018), higher BASDAI score (*p* = 0.042), and lower platelet count (*p* = 0.015) (Table 1). Those responders also had significant lower platelet count than nonresponders at 3 months after anti-TNF-α therapy (*p* = 0.015) (Figure 5).

**TABLE 1:** Differences in baseline clinical characteristics between responders and nonresponders.

Items	Responders (*N* = 21)	Nonresponders (*N* = 14)	*p* Value
Gender (male, %)	18 (85.7%)	12 (85.7%)	1.00
Age (year, mean ± SD)	32.86 ± 7.53	33.43 ± 10.73	0.85
Disease duration (year, mean ± SD)	8.10 ± 5.21	9.71 ± 5.07	0.37
ESR (median [Q25*–*Q75])	13 (6–30)	29 (14–49)	0.035
CRP (median [Q25*–*Q75])	2.70 (1.19–15.72)	8.50 (6.65–33.63)	0.018
ASDAS-CRP	2.73 ± 0.72	2.98 ± 0.84	0.35
BASDAI	4.68 ± 0.87	3.99 ± 1.05	0.042
Platelet (10^9^/L)	279.00 ± 51.10	333.40 ± 74.80	0.015

AS, ankylosing spondylitis; SD, standard deviation; data were shown as mean ± SD or median [Q25*–*Q75].

ROC analysis revealed that platelet count had a moderate role in predicting the response to anti-TNF-α therapy in AS patients with an area under the ROC curve (AUC) of 0.723 (95% CI 0.54–0.90) (Supplementary Figure S4). In the logistic regression analysis, lower platelet count was significantly associated with higher possibility of response to anti-TNF-α therapy in AS patients, and the OR of achieving response to anti-TNF-α therapy for each 50 × 10^9^/L decrement in platelet count was 2.26 (95% CI 1.06–4.82; *p* = 0.035). In the correlation analyses of those 35 AS patients, platelet count was correlated with ESR (*p* = 0.0001), CRP (*p* = 0.001), and ASDAS-CRP score (*p* = 0.002) but had no correlation with age (*p* = 0.54), gender (*p* = 0.39), and BASDAI (*p* = 0.72). In addition, ESR was intensively correlated with CRP (*p* < 0.001) and ASDAS-CRP (*p* < 0.001), and CRP was intensively correlated with ASDAS-CRP (*p* < 0.001), suggesting that those three variables were collinear. After adjusting for ASDAS-CRP score, lower platelet count was significantly associated with higher possibility of achieving good response to anti-TNF-α therapy (OR = 2.36, 95% CI 1.04–5.38; *p* = 0.04). After adjusting for ESR, lower platelet count was not significantly associated with the possibility of achieving good response to anti-TNF-α therapy (OR = 1.90, 95% CI 0.81–4.43; *p* = 0.14). After adjusting for CRP, lower platelet count was not significantly associated with the possibility of achieving good response to anti-TNF-α therapy (OR = 2.08, 95% CI 0.93–4.68; *p* = 0.08).

## Discussion

Although a number of clinical observational studies have investigated platelet changes in AS patients, definite conclusion on the clinical significance of platelet count in AS patients is still lacking. Therefore, we performed both a clinical observational study and a systematic review of relevant published studies to evaluate the clinical role of platelet count in AS patients. This study had several major findings. First, AS patients had higher platelet count than controls and anti-TNF-α therapy could reduce platelet count, which was further confirmed in the systematic review and meta-analysis. Second, platelet count was significantly and positively correlated with ESR, and it was moderately correlated with ASDAS-CRP score. Finally, those responders of anti-TNF-α therapy had significantly lower platelet count than nonresponders (*p* = 0.015) and platelet count was a biomarker of response to anti-TNF-α therapy in AS patients. The findings above expand our knowledge on the clinical significance of platelet count in AS patients.

Platelet parameters have been suggested as important markers of inflammation (Bilen et al., 2015; Kalelioglu et al., 2015; Akboga et al., 2016; Koupenova et al., 2018). Unlike other rheumatic disease such as SLE and Sjögren’s syndrome (SjS), AS is a rheumatic disease characterized by serious inflammatory responses (Vanaki et al., 2018; Vinker Shuster et al., 2018; Steiner et al., 2020). It has been clear that inflammation has a crucial role in the development of AS (Machado et al., 2016; Tseng et al., 2016; Ranganathan et al., 2017). CRP and ESR are commonly used biomarkers of inflammation in AS patients. The findings from our study showed obvious correlations of platelet count with inflammatory biomarkers such as CRP and ESR, suggesting that platelet number could reflect inflammation activity in AS patients. Therefore, apart from CRP and ESR, platelet number is another assistant inexpensive but reliable biomarker of inflammation severity in AS patients.

In this study of 35 AS patients, count was correlated with CRP and ASDAS-CRP, but it was not significantly correlated with BASDAI score. Platelet count probably has a relationship with ASDAS-CRP because it includes CRP, whereas BASDAI is an entirely patient-reported outcome and does not include inflammation, which is a possible explanation for the inconsistent correlations of platelet count with different disease activity markers in our study. Another study enrolling 122 AS patients found that there was no obvious difference in the platelet count among AS patients with different disease activity categorized by ASDAS-CRP score (Seng et al., 2018), which was inconsistent with the findings from our study. The evidence for the association between platelet count and disease activity in AS patients is still insufficient, and more future studies are needed to evaluate whether platelet count is a useful biomarker of disease activity in AS patients.

There were only two studies reporting data on the correlation between platelet count and CRP and three studies reporting data on the correlation between platelet count and BASDAI score, in which conflicting findings were reported (Figure 3A). There are two main possible explanations for the discrepancy among those studies. One main explanation is the low statistical power caused by the small sample size in those studies. Most studies recruited no more than 100 AS patients, which could result in low statistical power and subsequent inadequate estimations of the real correlations between platelet count and disease activity parameters such as CRP and BASDAI scores. Therefore, further studies with larger sample sizes are needed to provide a more reliable conclusion on the correlations between platelet count and disease activity parameters. Another possible explanation is the heterogeneity of AS patients among those included studies. AS patients from those studies had obvious differences in clinical characteristics such as disease activity, race, and treatment drugs, which could result in the discrepancy in the correlations between platelet count and disease activity parameters.

Findings from both this study and the meta-analysis confirm that anti-TNF-α therapy can reduce platelet count in AS patients (Figure 4). The platelet-lowering effect of anti-TNF-α therapy has been reported in patients with other autoimmune diseases such as psoriasis and IBD, and some of them even evolve into drug-induced side effects such as thrombocytopenia (Brunasso and Massone, 2009; Chen et al., 2011; Mares et al., 2011; Casanova et al., 2012). TNF-α has proved to promote megakaryocytic differentiation, indicating that anti-TNF-α therapy may have a direct inhibitory effect on platelet production by reducing megakaryocytic differentiation (Li et al., 1998). The risk of thrombocytopenia and subsequent major bleeding events should not be ignored in AS patients receiving anti-TNF-α therapy, and close monitoring of platelet counts may be recommended for those AS patients with lower platelet counts before anti-TNF-α therapy.

There are emerging studies suggesting the role of platelets as immunomodulators, and platelets can regulate the functions of immune cells such as T cells and macrophages (Dewi et al., 2020; Rolfes et al., 2020; Solomou et al., 2020). Many studies have proved that platelets have pathogenic roles in some autoimmune diseases such as RA and SLE (Boilard et al., 2010; Duffau et al., 2010; Boilard et al., 2012; Liu et al., 2020), but the potential role of platelets in the pathogenesis of AS has not received much attention. Apart from those studies assessing platelet counts in AS patients, there were several studies focusing on the abnormal changes in platelets in AS patients (Feltelius et al., 1986; Wang et al., 2008). An early study found that iron overload in platelets was associated with the inflammatory process in AS (Feltelius et al., 1986), and another clinical study suggested that AS patients had higher platelet activation than controls (Wang et al., 2008). Findings from this study and previously published studies have proved platelet parameters can be inflammatory markers in AS patients, but the correlation of platelets with immune dysfunction in AS patients is still unclear. So far, it is still uncertain whether platelets have a direct role in the pathophysiological processes of AS by regulating immune cells such as macrophages and T cells, which needs to be explored by future studies.

Previous studies have revealed that AS is associated with higher risk of cardiovascular pathologies (Bengtsson et al., 2017; Liew et al., 2018). The increased platelet count in AS patients may provide some explanations for the increased risk of cardiovascular diseases in AS patients. Increased platelet activation is a prothrombotic event and can promote the formation of atherosclerotic plaque (Hollan et al., 2013; Fuentes et al., 2019; Asada et al., 2020). The increased inflammation in AS patients with higher platelet count can also promote the development of cardiovascular injuries and atherosclerotic lesions (Agca et al., 2016; Maugeri et al., 2016; Dalbeni et al., 2017). Some recent studies have suggested a cardiovascular protective role of anti-TNF-α agents in patients with rheumatic diseases (Lee et al., 2018; Ntusi et al., 2018; Vegh et al., 2020), which may be at least partially explained by the platelet-suppressing role of anti-TNF-α agents. Anti-TNF-α agents have also proved to directly reduce platelet activation in patients with RA, which is a potential mechanism explaining the cardiovascular protective effect of anti-TNF-α therapy (Gasparyan and Kitas, 2016).

To the best of our knowledge, our study is the first one to identify elevated platelet count as a biomarker of poorer response to anti-TNF-α therapy in AS patients. The result must be interpreted with caution because the finding is from a single-center study and its sample size is not big enough. More studies with large number of participants are needed to verify the findings in this study. Moreover, there are conflicting findings in the outcomes of multivariate logistic regression analyses when adjusting for different disease activity markers, which is likely to be caused by the low statistical power in our study. More studies are needed to provide further evidence for platelet count as a biomarker of response to anti-TNF-α therapy in AS patients.

In summary, this study reveals that platelet count is related to inflammation severity of AS patients, and elevated platelet count is a promising biomarker of poorer response to anti-TNF-α therapy in AS patients. More studies are needed to further assess the impact of platelet count on treatment outcomes in AS patients, and studies elucidating the pathogenic role of platelet in AS are warranted.

## Data Availability Statement

All data sets presented in this study are included in the article/Supplementary Material.

## Ethics Statement

The studies involving human participants were reviewed and approved by Ethics Committee of the First Affiliated Hospital of Xiamen University. The patients/participants provided their written informed consent to participate in this study. Written informed consent was obtained from the individual(s) for the publication of any potentially identifiable images or data included in this article.

## Author Contributions

HQ, YL, and GS contributed to conception and design; HQ and BW performed the experiments and statistical analysis; RC rechecked the data and revised the manuscript; XY and SC helped for the data statistical analysis and wrote section of the manuscript. All authors contributed to manuscript revision, and read and approved the submitted version.

## Funding

This work was supported by grants from the National Natural Science Foundation of China (Grant Nos. 81971536, 81601384, and 81971496).

## Conflict of Interest

The authors declare that the research was conducted in the absence of any commercial or financial relationships that could be construed as a potential conflict of interest.
