# Supplementary figures and images for: Associations of Platelet Count with Inflammation and Response to Anti-TNF-α Therapy in Patients with Ankylosing Spondylitis
Source: Front Pharmacol. 2020 Nov 6;11:559593. doi: 10.3389/fphar.2020.559593 (PMC7741170; doi:10.3389/fphar.2020.559593)

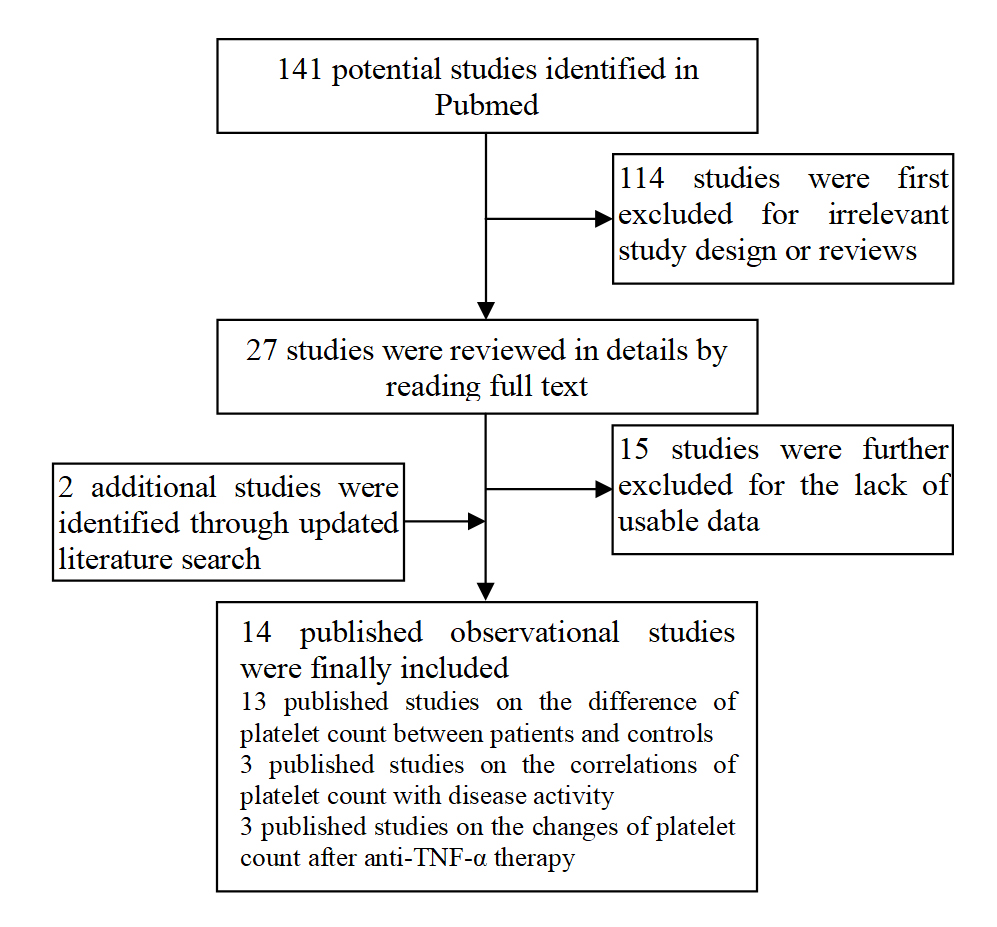

Supplement: Supplementary file 1 [file Image1_v1.JPEG]

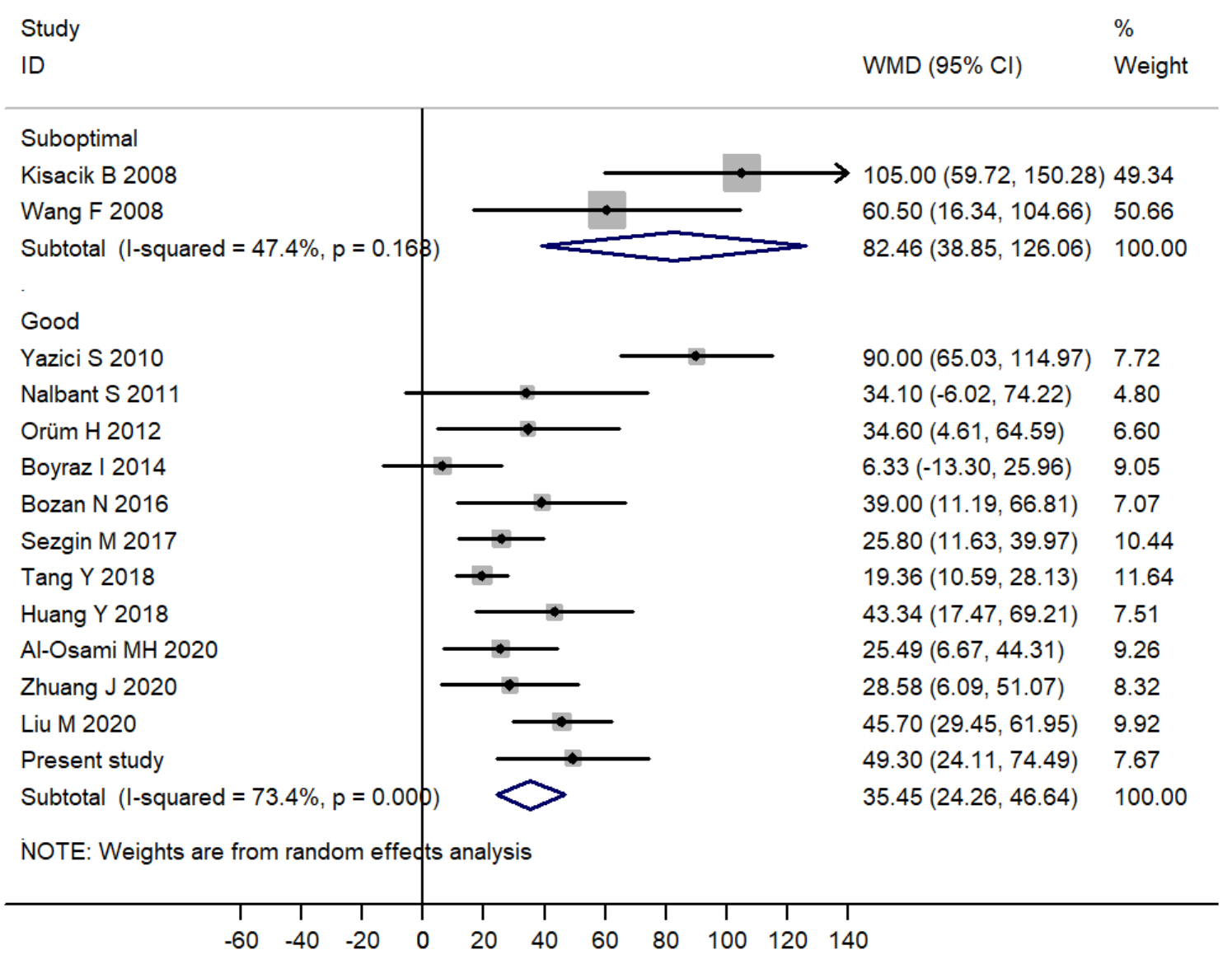

Supplement: Supplementary file 2 [file Image2_v1.JPEG]

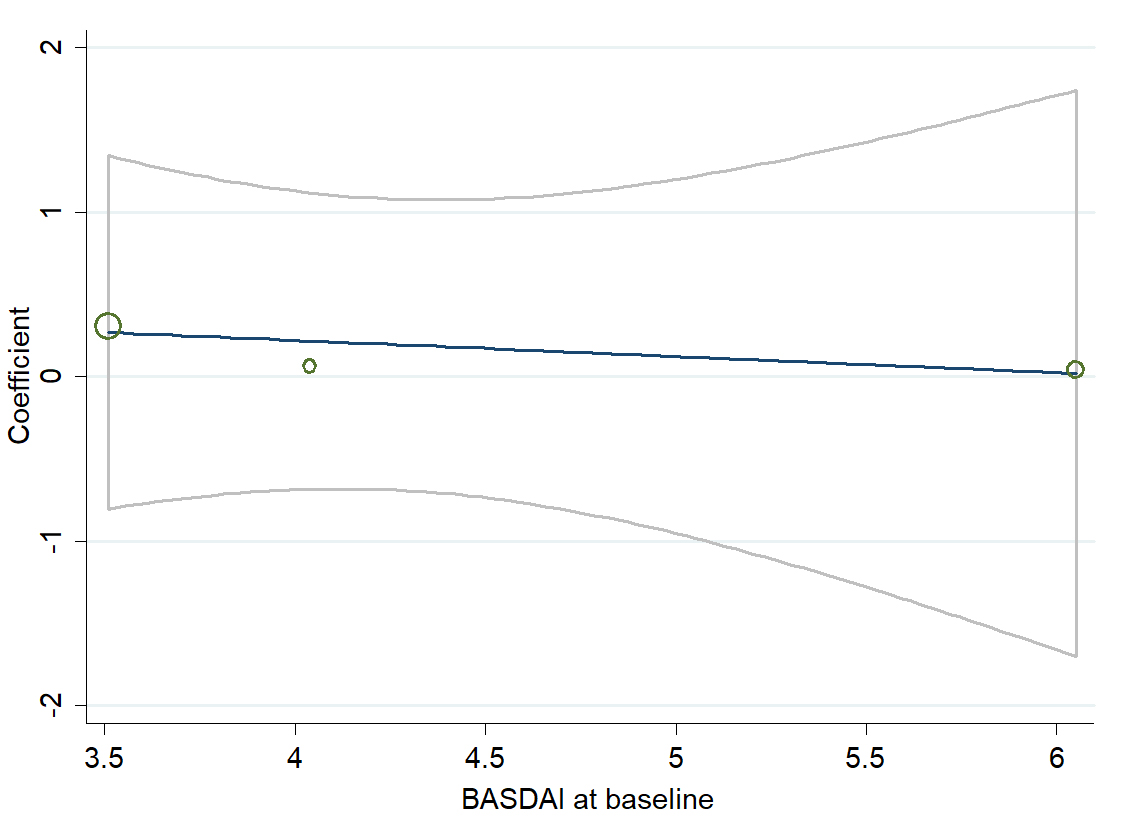

Supplement: Supplementary file 3 [file Image3_v1.JPEG]

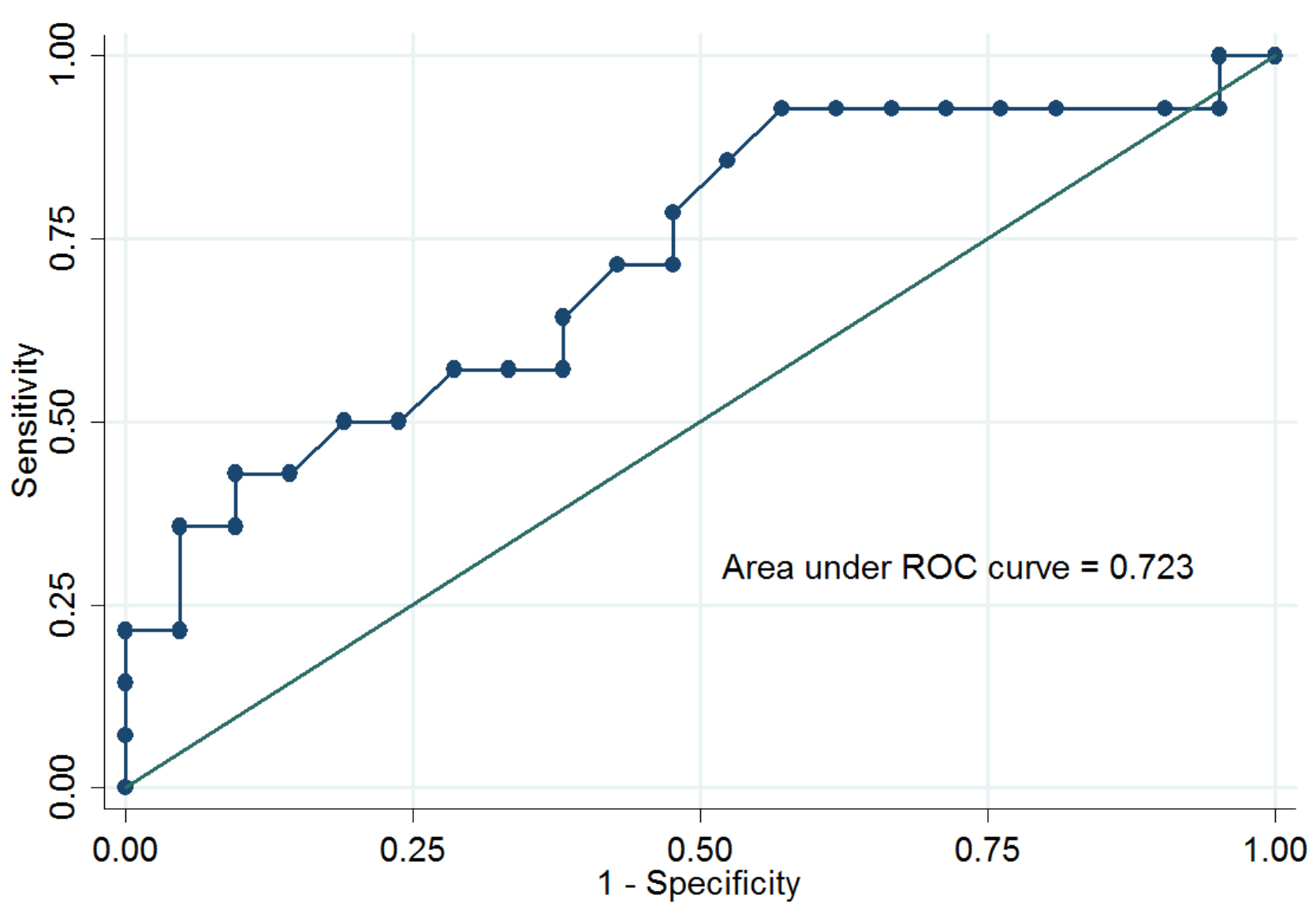

Supplement: Supplementary file 4 [file Image4_v1.JPEG]
